# Modulating ascorbic acid levels to optimize somatic embryogenesis in *Picea abies* (L.) H. Karst. Insights into oxidative stress and endogenous phytohormones regulation

**DOI:** 10.3389/fpls.2024.1372764

**Published:** 2024-06-05

**Authors:** Teresa Hazubska-Przybył, Agata Obarska, Agata Konecka, Joanna Kijowska-Oberc, Mikołaj Krzysztof Wawrzyniak, Alicja Piotrowska-Niczyporuk, Aleksandra Maria Staszak, Ewelina Ratajczak

**Affiliations:** ^1^ Institute of Dendrology, Polish Academy of Sciences, Kórnik, Poland; ^2^ Institute of Forest Sciences, Warsaw University of Life Science, Warsaw, Poland; ^3^ Department of Plant Biology and Ecology, University of Bialystok, Bialystok, Poland

**Keywords:** ASA, conifers, hydrogen peroxide, cytokinins, brassinosteroids

## Abstract

Global warming has adversely affected *Picea abies* (L.) H. Karst. forests in Europe, prompting the need for innovative forest-breeding strategies. Somatic embryogenesis (SE) offers promise but requires protocol refinement. Understanding the molecular mechanisms governing somatic embryo development is essential, as oxidative stress plays a crucial role in SE regulation. Ascorbic acid (ASA), is a vital antioxidant that can potentially control oxidative stress. In the present study, we normalized ASA concentrations in induction and proliferation media to enhance embryogenic tissue (ET) regeneration and proliferation capacity of mature explants. The media were supplemented with ASA at 0 mg l^−1^, 25 mg l^−1^, 50 mg l^−1^, 100 mg l^−1^, and 200 mg l^−1^. The accumulation of hydrogen peroxide (H_2_O_2_) and endogenous phytohormones, including auxins, cytokinins, brassinosteroids, abscisic acid, and gibberellin, was measured in non-embryonic calli and ET. Subsequently, their impact on ET induction and multiplication was analyzed. Our results demonstrate that application of ASA at concentrations of 25 mg l^−1^ and 200 mg l^−1^ led to increased H_2_O_2_ levels, potentially inducing oxidative stress while simultaneously reducing the levels of all endohormone groups. Notably, the highest ET induction frequency (approximately 70%) was observed for ASA at 50 mg l^−1^. These findings will enhance SE induction procedures, particularly in more resistant explants, underscoring the significance of ASA application to culture media.

## Introduction

Currently, spruce forests are in decline due to climate change (e.g., droughts, fires, and pathogen outbreaks), especially in Central Europe (Caudullo et al., 2016; Wojnicka-Półtorak et al., 2023). Global warming affects the frequency of extreme weather events and disrupts thermal signals, transforming the stages of seed production, dormancy, and germination (Kijowska-Oberc et al., 2020). Norway spruce (*Picea abies* (L.) H. Karst.) is one of the species most threatened by the effects of climate change on forest-forming species. Dyderski et al. (2018) predicted, based on modeling analyses, that its distribution in Europe will gradually decrease in the near future. To prevent the loss of genetic diversity and wood raw material for the paper, construction, and furniture industries, it would be beneficial to integrate the somatic embryogenesis (SE) technique into forestry practice (Hazubska-Przybył et al., 2022). However, existing protocols still need to be optimized and standardized to be universally applicable (Denchev and Grossnickle, 2019). It is believed that in-depth, more detailed biochemical and molecular studies on the interaction of specific factors at different stages of somatic embryonic development will bring scientists closer to this goal (Trontin et al., 2016). SE is potentially the most effective *in vitro* method for the large-scale production of coniferous somatic seedlings. Although numerous SE-based propagation protocols have been developed, the implementation of SE in breeding practices remains limited (Hazubska-Przybył and Bojarczuk, 2016; Denchev and Grossnickle, 2019). Norway spruce is a model species for coniferous SE phenomenon research and is an important tree for commercial production (Lelu-Walter et al., 2013).

Some studies have reported that ascorbic acid (ASA) can have a positive effect on SE in spruce at the physiological level (Stasolla and Yeung, 2007; Zheleznichenko and Novikova, 2017). However, they mainly refer to the maturation and germination stages of the somatic embryos. We focused on SE induction from mature zygotic embryos and proliferation of the obtained embryogenic tissue (ET). SE induction using older explants remains problematic (Ilashi 2016 after 12). In turn, the loss of induced ETs, which are the source of immature somatic embryos (proembryos) and consequently the genetic diversity provided by ET lines (genotypes), is another major problem limiting the large-scale use of SE (Hazubska-Przybył et al., 2020). ASA is a natural plant compound involved in the regulation of basic cellular processes (Stasolla and Yeung, 2007; Akram et al., 2017) as a strong antioxidant that prevents oxidative damage in cells. It acts as a cofactor for dioxygenases in the biosynthesis of phytohormones such as ethylene, gibberellins (gibberellic acid; GA), auxins (indole-3-acetic acid; IAA), and abscisic acid (ABA), and participates in their signal transduction pathways. ASA is also involved in metabolic crosstalk between redox-regulated pathways and hormones (Ortiz-Espín et al., 2017).

Our main objective was to assess whether exogenous ASA supplementation modulates oxidative stress and endogenous phytohormone activity, and whether it consequently improves SE efficiency during the first stages of somatic embryo development. This new information will also help increase the quality of mature embryos and their germination and conversion rates into somatic seedlings. Therefore, the aim of this study was to examine whether there is any relationship between ASA applied to media and both the hydrogen peroxide (H_2_O_2_) level and the endogenous phytohormone activity during both steps of SE, and whether its application to media would allow for improvement of ET induction and proliferation yield.

## Methods

### Plant material

ETs were induced from mature zygotic embryos excised from seeds collected from cones harvested in 2020 at the “Zwierzyniec” Experimental Forest in Kórnik (52°15′N, 17°04′E). Seed sterilization, ET induction, and proliferation were carried out according to Hazubska-Przybył et al. (2020) with modifications.

### ET induction and proliferation

During the induction phase (Experiment I), explants were treated with ASA at 0 mg l^−1^, 25 mg l^−1^, 50 mg l^−1^, 100 mg l^−1^, and 200 mg l^−1^. Thirty explants (five per Petri dish × six) for each ASA dose were used. After a few weeks, the induction of a specific flocculent white embryogenic tissue (see 15) containing early somatic embryonic stages, followed by some of the yellow-green, non-embryogenic explants with a compact structure. The ET induction frequency (%) was assessed after 8 weeks of incubation in the dark at 22°C. The same set of ASA concentrations was tested during the ET proliferation step. One selected ET line (IC 94) multiplied by an ASA-free medium was used in Experiment II. ET mass gain was estimated after a 7-day incubation under the same conditions as those during SE induction. Ten pieces of ET (with an average weight of approximately 250 mg per piece) per ASA treatment were used for ET mass gain estimation.

### H_2_O_2_ content measurement

The H_2_O_2_ content in non-embryogenic calli (Experiment I) and in the proliferated ET (Experiment II) was measured according to the Sagisaka (1976) method. After grinding, the samples were placed in liquid nitrogen and homogenized with 5 ml of 5% (w/v) trichloroacetic acid (TCA) and 10 mM ethylenediaminetetraacetic acid (EDTA). The resulting homogenate was centrifuged at 20,000×*g* and 4°C for 20 min. The entire volume of the supernatant was analyzed.

### Phytohormone content measurement

The phytohormone-related analyte profiling method described by Šimura et al. (2018) was used to assess the phytohormone content in the fresh plant material. Each sample weighed approximately 200 mg.

Measurements of both H_2_O_2_ and phytohormone contents were performed for non-embryogenic calli (after 8 weeks of explant incubation on induction media) and ET (after a 7-day proliferation cycle).

### Statistical analysis

All analyses were performed using the statistical language R (R Core Team, 2020). One-way analysis of variance (ANOVA) was used to assess the effect of ASA concentration on all parameters studied (ET induction, ET mass gain, H_2_O_2_, and phytohormone content). Assumptions of normality and homogeneity of variance were checked before the analysis. Significant differences between means were assessed using Tukey’s *post hoc* test (ET induction and proliferation) or Duncan’s test (H_2_O_2_ and phytohormones).

## Results

SE is a complex embryonic development process controlled by numerous exogenous and endogenous factors. One of the most important factors is oxidative stress generated under artificial *in vitro* culture conditions, which can modulate the SE course. Therefore, in this study, we focused on assessing how exogenously applied ASA can influence the H_2_O_2_ pool and endogenous phytohormone activity in cells, and whether this influence is related to the efficiency of the SE induction process and multiplication of ET.

### Experiment I. SE induction stage

**Figure 1:**
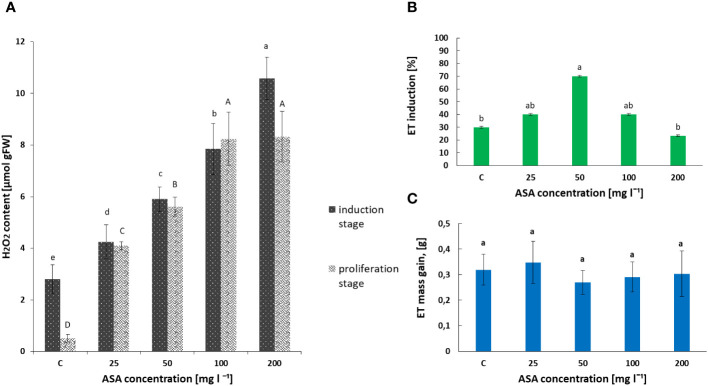
Effect of ASA concentration (C = 0 mg l^−1^–200 mg l^−1^) on hydrogen peroxide (H_2_O_2_) content **(A)** and *P. abies* ET induction **(B)** and proliferation **(C)** levels. For **(A)** results, one-way ANOVA, followed by *post hoc* Duncan’s test, while for **(B, C)** results, one-way ANOVA, followed by *post-hoc* Tukey’s test were carried out; means with the same letter are not significantly different at p <0.05. Means ± SEs.

Our biochemical analysis revealed that the H_2_O_2_ content in the non-embryogenic callus, which developed after 8 weeks of treatment of *P. abies* explants with ASA at 0 mg l^−1^–200 mg l^−1^, increased significantly with an increase in the concentration of this antioxidant in the induction medium (**Figure 1A**). The lowest value was observed for the control variant without ASA supplementation and the highest value was observed for 200 mg l^−1^ ASA.

**Figure 2:**
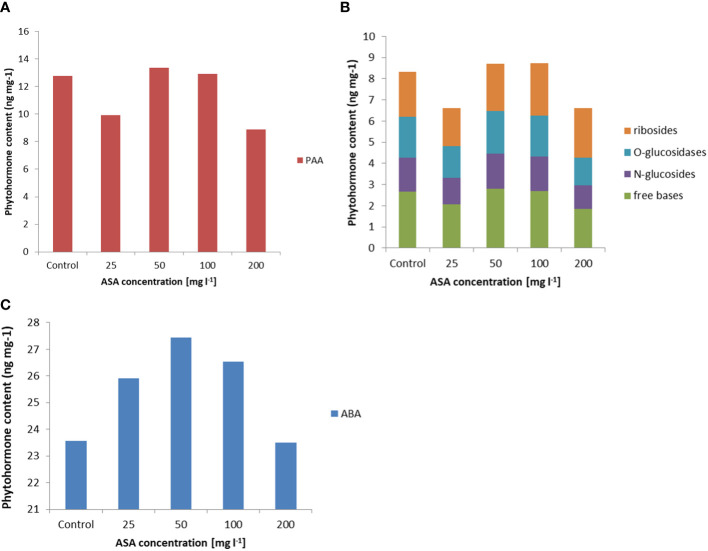
Endogenous phytohormone content in non-embryogenic calli after 8 weeks of incubation of explants on ½ LM induction medium supplemented with ASA at 0 mgl^-1^ –200 mgl^-1^. **(A)** Auxins, **(B)** CKs, and **(C)** ABA. For each result, one-way ANOVA, followed by *post-hoc* Duncan’s test, was carried out; means with the same letter are not significantly different at p <0.05. Means ± SEs.

In turn, the analysis of phytohormones based on simultaneous LC−MS/MS-based profiling led to the identification of 34 endogenous compounds in these calli. The identified compounds were auxins (two forms), cytokinins (CKs; 22 forms), ABA, GA3 (one form), and brassinosteroids (BRs; eight forms). Concentrations of auxin (PAA), some CKs, and ABA varied considerably in this plant material, depending on the ASA concentration in the induction medium (see **Figure 2** and **Supplementary Table S1**). Of the two endogenous auxin forms detected in non-embryogenic calli, only non-indole phenolic compound (PAA) activity was significantly affected by ASA dose. As it is a compound with weak auxin activity, its content ranged from 8.871 ng mg^−1^ to 13.370 ng mg^−1^ (**Figure 2A**). In the CK group, free bases (bioactive forms), N-glucosides (putatively irreversible deactivation forms), O-glucosidases (reversible storage forms), and ribosides (transport forms) were identified (**Figure 2B**). CKs were detected at lower concentrations than auxins, and the highest amounts were free bases and ribosides. Their content was determined by the concentration of exogenous ASA in the medium. The highest amounts, but comparable to those of the control variant, were observed in the presence of 50 mg l^−1^ and 100 mg l^−1^ ASA. Similar relationships were observed for the auxins. However, at both the lowest and highest antioxidant doses tested, the CK content was significantly lower (see **Figures 2A, B**). In turn, ABA was present in higher amounts in non-embryogenic calli than in auxin and CKs (**Figure 2C**), with the highest concentration (27.447 ng mg^−1^) observed when 50 mg l^−1^ ASA was applied to the medium. In the control treatment, its content was significantly lower at 23.567 ng mg^−1^. These results are consistent with the frequency of ET induction from mature zygotic embryos at particular ASA doses (**Figure 1B**). Exogenous ASA clearly determined the ET regeneration rate. The best result (approximately 70%) was obtained when 50 mg l^−1^ ASA was applied to the medium. The lowest result (approximately 23%) was recorded for 200 mg l^−1^ ASA in the medium. An increase in ASA concentration above this level resulted in a gradual decrease in the ability of the explants to produce ET. This means that a higher concentration of ASA generates a higher concentration of H_2_O_2_ in cultivated explants. Thus, the observed excessive accumulation of H_2_O_2_ in 8-week-old calli may be related to an increase in the level of oxidative stress, which in turn limits SE efficiency at the ET induction stage. However, the use of ASA in the medium at a concentration of 50 mg l^−1^ indicated that the interaction between this antioxidant and H_2_O_2_ was the most balanced, resulting in the most beneficial effect on the induction of ET from the tested explants.

### Experiment II. SE proliferation stage

**Figure 3:**
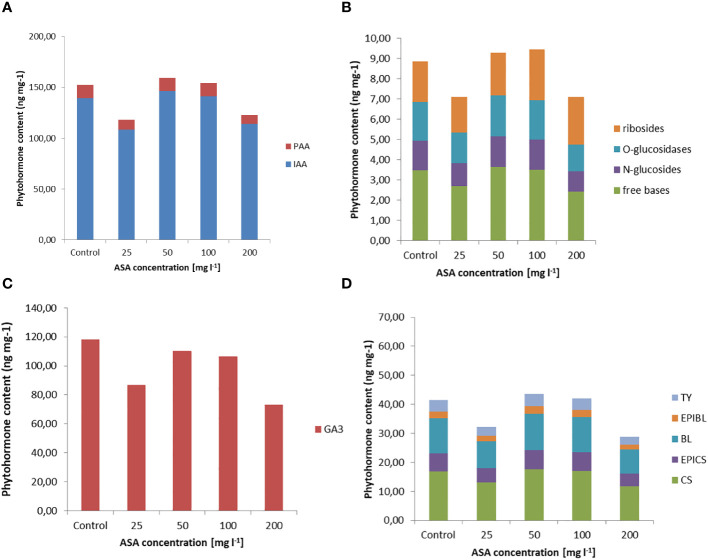
Endogenous phytohormone content in ET after 7 days of incubation on ½ LM proliferation medium supplemented with ASA at 0 mgl^-1^ –200 mgl^-1^. **(A)** Auxins, **(B)** CKs, **(C)** GA3, and **(D)** BRs. For each result, one-way ANOVA, followed by *post-hoc* Duncan’s test, was carried out; means with the same letter are not significantly different at p <0.05. Means ± SEs.

Exogenously applied ASA also determined the H_2_O_2_ content in induced and multiplied ET after 7 days of treatment. The highest values were obtained for ASA at 100 mg l^−1^ and 200 mg l^−1^ (**Figure 1A**). The same set of 34 phytohormones identified in non-embryogenic calli was also found in ET (**Figure 3** and **Supplementary Table S1**). Quantitatively, IAA (146.20 ng mg^−1^ for ASA at 50 mg l^−1^) and GA3 (~.118.22 ng mg^−1^ for the control variant) dominated ET (**Figures 3A, C**). PAA concentration was comparable to that during ET induction (between 8 ng mg^−1^ and 14 ng mg^−1^; **Figures 2A**, **3A**). Our analysis showed a slightly higher concentration of free-based CKs compared to the previous SE stage, whereas for the other stages, their content was similar in both non-embryogenic calli and ET (**Figure 3B**). Furthermore, during ET proliferation, several phytohormones were identified in the BR group (**Supplementary Table S1**). The contents of five compounds, castasterone (CS), 24-epicastasterone (EPICS), brassinolide (BL), typhasterol (TY), and 24-epibrasinolide (EPIBL), were significantly dependent on exogenous ASA treatment (**Figure 3D**). In each of the identified groups of phytohormones, a similar relationship was observed between their activity and the dose of ASA in the proliferation medium, and a significant decrease in their activity was observed in the presence of ASA at 25 mg l^−1^ and 200 mg l^−1^ (**Figure 1C**). The highest ET mass gain (approximately 0.35 g) was observed for ASA (25 mg l^−1^). The lowest result (approximately 0.27 g) was observed after 50 mg l^−1^ ASA treatment. Finally, we found that exogenous ASA did not significantly affect ET proliferation after 7 days of treatment.

## Discussion

ASA is one of the most powerful antioxidant molecules involved in quenching the reactive forms of molecular oxygen. The ascorbate–glutathione pair regulates plant development (Akram et al., 2017). We found that exogenously applied ASA modulated H_2_O_2_ and some endogenous phytohormone contents in the non-embryonic calli of *P. abies*, which developed after 8 weeks of incubation of explants (mature zygotic embryos). A clear relationship between ASA dose and H_2_O_2_ content was also observed during ET proliferation. According to Akram et al. (2017), the addition of ASA to the culture medium can modulate the tissue culture environment. However, it is also known that this molecule has a very short lifetime under in vitro culture (only hours) (Feng et al., 1977) and very low absorption capacity from plant cell – see (Gallie, 2013) and references within. After a few hours, ASA is oxidised into DHA (dehydroascorbate), which has a much higher affinity for absorption by plants. ASA itself might be very toxic to plant cells (as also shown in this manuscript), with a very narrow threshold. On the other hand, DHA can be of more benefit to plant cells (Chavan et al., 2022) and references within) and is reconverted to ASA in the cytoplasm.

ASA may be involved in embryogenesis (Arrigoni et al., 1992) by participating in the activation of various antioxidant enzymes. Developing embryos are sensitive to intracellular H_2_O_2_ concentrations (Yang et al., 2021), which can generate oxidative stress at higher concentrations and thus affect vital plant functions *in vitro* (Välimäki et al., 2021). On the other hand, ASA, as a strong antioxidant, prevents unfavorable H_2_O_2_ levels in cells, protecting them from damage. H_2_O_2_ at appropriate levels can act as a secondary messenger in signal transduction and regulate gene expression and protein synthesis (Smirnoff and Arnaud, 2019). Hence, it may have a positive impact on somatic embryo development. It has been shown that ROS-associated antioxidants can determine embryonic growth at the early stages of development (Wang and Yao, 2023).

ASA is closely linked to ascorbate-hormone crosstalk and is involved in the regulation of plant development and growth (Akram et al., 2017). According to Sadak et al. (2013), foliar treatment with ASA:citric acid resulted in an increase in GA, IAA, zeatin (Z), and BR content in wheat, while the ABA content significantly decreased. Most of these phytohormones also play important roles in conifer SE (Vondrakova et al., 2018). Therefore, understanding the interactions among exogenous ASA, ROS, and endohormones would enable the development of more efficient micropropagation protocols.

In conifers, SE induction and proliferation are highly dependent on exogenous auxin and CK supplementation, as both determine the acquisition of totipotency by explant cells (Feher et al., 2008) and trigger SE. They regulate cell division and differentiation in plant cells (Teixeira da Silva and Malabadi, 2012), leading to the formation of early stage somatic embryos. Although the role of auxins in conifer SE is well documented (Isah, 2016), the involvement of other endohormones, e.g., CKs, GA, and BRs, at different stages of SE is poorly understood (Vondrakova et al., 2018). Auxins and CKs interact with each other, thereby affecting the activity of other phytohormones. However, some effects of CKs may be antagonized by ABA. CKs also promote ethylene biosynthesis under *in vitro* conditions (Gaspar et al., 1996). In turn, BRs regulate the expression of hundreds of genes, affect the activities of numerous metabolic pathways, and control the developmental programs, leading to morphogenesis. Therefore, their association with SE in coniferous species is highly probable (Pullman and Bucalo, 2011).

In our study, analysis of phytohormone content revealed the presence of 34 compounds, both in calli and ET-proliferated cell lines (**Supplementary Material Table 1**). A marked decrease in phytohormones, including those involved in the conifer SE process, such as IAA, PAA, cis-zeatin-ribose (cZR), dihydrozeatin (DHZ), and ABA (Klimaszewska et al., 2009; Vondrakova et al., 2018), in the presence of 25 mg l^−1^ and 200 mg l^−1^ ASA was observed. We found that this antioxidant significantly modified the activity of some endogenous phytohormones identified, depending on the level of supplementation. Hence, we assume that it may be involved in controlling the direction of embryonic development during the early SE steps. The increased frequency of embryogenesis induction and proper morphological development of early somatic embryos determine the high biological diversity of potentially high-quality planting material obtained by SE. Vondrakova et al. (2018) showed that phytohormones play a key role in regulation of *P. abies* somatic embryo development at particular SE stages. In our work, we focused on the first two SE steps (induction and proliferation of ET), as such analyses have rarely been undertaken. Recently, Tretyakova et al. (2019) performed an immunoassay of plant hormones using extracts from explants and long-proliferating ET lines of *Larix sibirica*.

Our study showed that ASA concentration affected ET induction frequency in mature zygotic embryos of *P. abies*. The application of 50 mg l^−1^ ASA to the medium was the highest promoting dose for this explant type. This relationship was not observed during ET multiplication. To date, only a few publications have shown at the physiological level that ASA may be involved in promoting the SE process in conifers (Stasolla and Yeung, 2007; Pullman et al., 2015; Zheleznichenko and Novikova, 2017). According to Zheleznichenko and Novinkova (Zheleznichenko and Novikova, 2017), this antioxidant stimulates the initial steps of somatic embryo development in *Picea pungens* Engelmann. Stasolla and Yeung (2007) reported a positive effect on somatic embryo formation in *Picea glauca* (Moench) Voss. The induction and proliferation stages of *P. pungens* were promoted even when the basal medium was supplemented with 300 mg l^−1^ ASA (Zheleznichenko and Novikova, 2017). On the other hand, Malabadi and Van Staden (2005) reported a negative effect of ASA at a concentration of 0.01%–0.5% on SE induction from vegetative shoot apices of *Pinus patula*. In our study, the highest doses of ASA (>100 mg l^−1^) reduced SE induction but did not limit ET proliferation.

**SCHEME 1:**
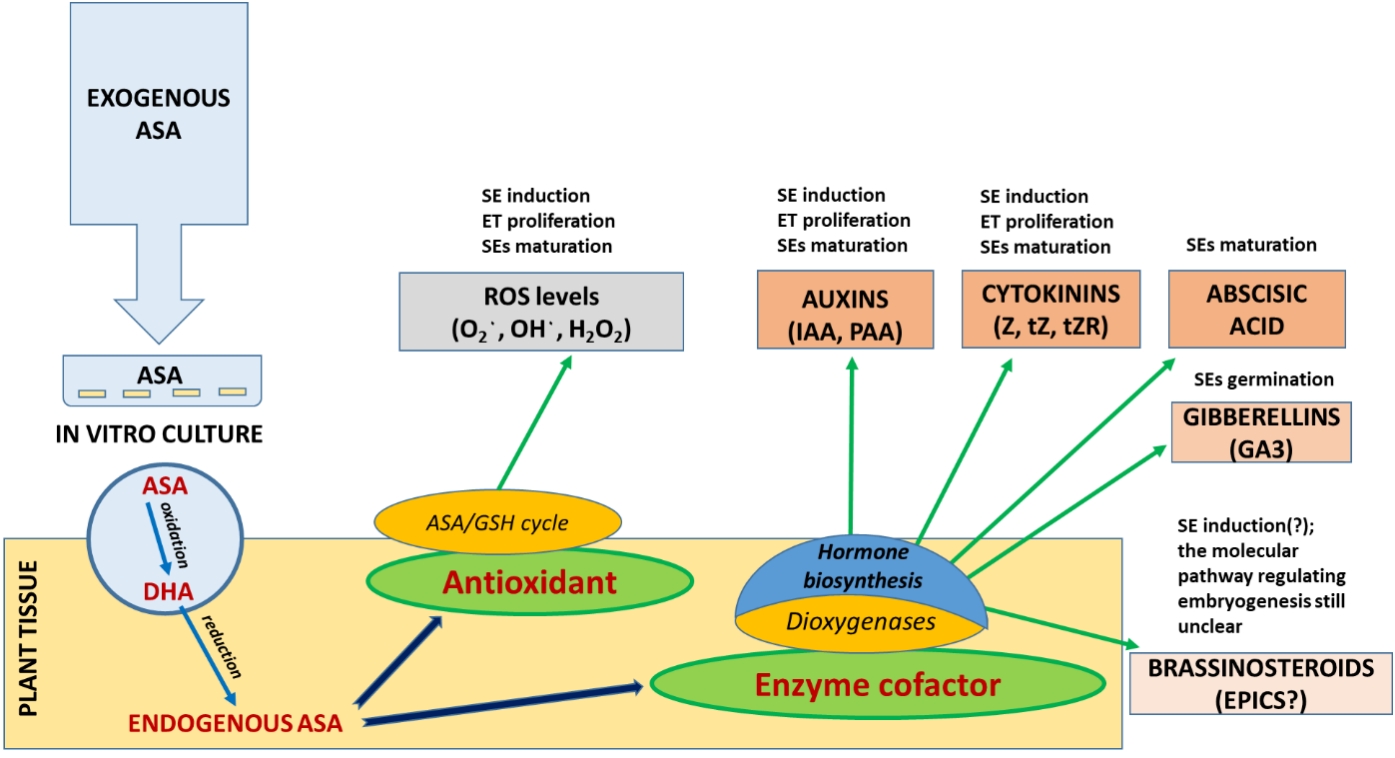
Regulation of the level of oxidative stress and phytohormone activity in the first stages of SE (ET induction and proliferation) of *P. abies* by exogenous supplementation of culture media with ascorbic acid (ASA).

The aim of the study was not only to elucidate how embryos develop at a physiological and biochemical level based on the analysis of endogenous hormonal profiles but also to identify opportunities to manipulate the environment of the tissue culture by ASA application to improve SE efficiency, starting with SE induction (**Scheme 1** ). The preliminary findings from this study will serve as a foundation for more comprehensive molecular-level investigations into the intricate crosstalk between signaling pathways at the signal transduction level, impacting these regulatory processes. A comprehensive understanding of these interconnections will be instrumental in the enhancement and innovation of SE induction protocols, particularly for more resistant explants.

## Data availability statement

The raw data supporting the conclusions of this article will be made available by the authors, without undue reservation.

## Author contributions

TH-P: Writing – original draft, Writing – review & editing. AO: Writing – review & editing. AK: Writing – review & editing. JK-O: Writing – original draft, Writing – review & editing. MW: Writing – original draft, Writing – review & editing. AP-N: Writing – original draft, Writing – review & editing. AS: Writing – original draft, Writing – review & editing. ER: Writing – original draft, Writing – review & editing.

## Supplementary material

Supplementary Table 1 The content of endogenous phytohormones in plant material during the induction and proliferation of ET *Picea abies*, after supplementation of the media with ASA, at 0–200 mg l^-1^.
