# Supplementary material for: Modulating ascorbic acid levels to optimize somatic embryogenesis in Picea abies (L.) H. Karst. Insights into oxidative stress and endogenous phytohormones regulation
Source: Front Plant Sci. 2024 Jun 5;15:1372764. doi: 10.3389/fpls.2024.1372764 (PMC11188323; doi:10.3389/fpls.2024.1372764)
Supplement: Supplementary Table 1 — The content of endogenous phytohormones in plant material during the induction and proliferation of ET Picea abies, after supplementation of the media with ASA, at 0–200 mg l-1. [file DataSheet_1.pdf]

Table S1. The content of endogenous phytohormones in plant material during the induction and proliferation of ET *Picea abies*, after supplementation of the media with ASA, at 0-200 mg l^{-1}.

	Non-Embryonic tissue					Embryonic tissue				
	ASA concentration (mg l-1)					ASA concentration (mg l-1)				
Hormone (ng mg -1)	0	25	50	100	200	0	25	50	100	200
trans-Zeatin-Riboside (tZR)	2,38±0,18b	1,85±0,3ab	2,49±0,14a	2,41±0,04ab	2,29±0,18a	ns	ns	ns	ns	ns
trans-Zeatin-9-Glucoside (tZ9G)	1,78±0,04b	1,37±0,23ab	1,86±0,34a	1,8±0,47ab	1,26±0,43a	ns	ns	ns	ns	ns
trans-Zeatin-7-Glucoside (tZ9G)	1,16±0,09b	0,91±0,13ab	1,22±0,05a	1,18±0,19ab	0,81±0,15a	1,16±0,09a	0,91±0,13b	1,22±0,05a	1,18±0,19a	0,81±0,15b
trans-Zeatin-O-Glucoside (tZOG)	1,73±0,14b	1,35±0,24ab	1,81±0,13a	1,75±0,29ab	1,19±0,16a	1,73±0,14a	1,35±0,24b	1,81±0,13a	1,75±0,29a	1,19±0,16b
trans-Zeatin-O-Glucoside Riboside (tZROG)	2,12±0,18b	1,65±0,3ab	2,22±0,18a	2,14±0,36ab	1,46±0,18a	2,12±0,18ab	1,65±0,3b	2,22±0,18a	2,14±0,36a	1,46±0,18b
cis-Zeatin-Riboside (cZR)	2,23±0,2c	2,14±0,29abc	2,33±0,1ab	3,2±0,93bc	3,47±0,23a	2,23±0,2b	2,14±0,29b	2,33±0,1b	3,2±0,93a	3,47±0,23a
cis-Zeatin-O-Glucoside Riboside (cZROG)	ns	ns	ns	ns	ns	1,26±0,16ab	0,97±0,11bc	1,32±0,1a	1,27±0,24ab	0,88±0,21c
Dihydrozeatin (DHZ)	ns	ns	ns	ns	ns	1,97±0,15a	1,53±0,22b	2,07±0,08a	2±0,33a	1,37±0,24b
Dihydrozeatin Riboside (DHZR)	1,77±0,18b	1,39±0,29ab	1,86±0,21a	1,8±0,33b	1,22±0,11a	1,77±0,18ab	1,39±0,29bc	1,86±0,21a	1,8±0,33ab	1,22±0,11c
Dihydrozeatin-7-Glucoside (DHZ7G)	1,04±0,08c	0,81±0,13abc	1,09±0,06ab	1,05±0,17bc	0,72±0,11a	1,04±0,08a	0,81±0,13b	1,09±0,06a	1,05±0,17a	0,72±0,11b
N6-Isopentenyladenine (iP)	ns	ns	ns	ns	ns	3,96±0,54a	3,06±0,36ab	4,14±0,37a	4±0,78a	2,77±0,7b
N6-sopentenyladenosine (iPR)	ns	ns	ns	ns	ns	4,47±0,39a	3,47±0,45b	4,68±0,19a	4,52±0,75a	3,1±0,61b

N6-Isopentenyladenine-7-Glucoside (iP7G)	2,41±0,19c	1,88±0,33abc	2,52±0,19ab	2,44±0,41bc	1,66±0,22a	2,41±0,19a	1,88±0,33b	2,52±0,19a	2,44±0,41a	1,66±0,22b
o-topolin (oT)	0,86±0,11b	0,68±0,16ab	0,91±0,13a	0,88±0,17ab	0,59±0,04a	ns	ns	ns	ns	ns
Indole-3-acetic acid (IAA)	ns	ns	ns	ns	ns	139,61±12,7a	108,34±13,7b	146,2±6,21a	141,29±23,84a	113,85±2,9b
Phenylacetic acid (PAA)	12,77±1,17c	9,91±1,25abc	13,37±0,58ab	12,92±2,18bc	8,87±1,8a	12,77±1,17a	9,91±1,25b	13,37±0,58a	12,92±2,18a	8,87±1,8b
Abscisic Acid (ABA)	23,57±6,03b	25,9±7,31ab	27,45±1,31a	26,53±4,55b	23,49±3,83a	ns	ns	ns	ns	ns
Giberelin (GA3)	ns	ns	ns	ns	ns	118,22±0,66a	86,66±10,51bc	110,22±6,57ab	106,54±18,91ab	73,36±16,52c
Castasterone (CS)	ns	ns	ns	ns	ns	16,87±1,53a	13,09±1,66b	17,66±0,75a	17,07±2,88a	11,72±2,37b
24-Epicastasterone (EPICS)	ns	ns	ns	ns	ns	6,3±0,52a	4,9±0,66b	6,6±0,25a	6,38±1,05a	4,37±0,82b
Brassinolide (BL)	ns	ns	ns	ns	ns	11,97±1,1a	9,29±1,17b	12,54±0,54a	12,12±2,05a	8,32±1,7b
24-Epibrassinolide (EPIBL)	ns	ns	ns	ns	ns	2,37±0,3ab	1,84±0,22abc	2,48±0,2a	2,4±0,45ab	1,66±0,41c
Typhasterol (TY)	ns	ns	ns	ns	ns	4±0,3a	3,12±0,5b	4,2±0,23a	4,06±0,66a	2,77±0,42b

Mean ± standard deviation Duncan Test
